# The endocannabinoid system in cancer biology: a mini-review of mechanisms and therapeutic potential

**DOI:** 10.3389/or.2025.1573797

**Published:** 2025-04-30

**Authors:** Kaio Cezar Rodrigues Salum, Gabriel Brendo Alves Miranda, Alessandra Lima Dias, João Regis Ivar Carneiro, Patrícia Torres Bozza, Ana Carolina Proença da Fonseca, Tamara Silva

**Affiliations:** ^1^ Clementino Fraga Filho University Hospital, Federal University of Rio de Janeiro, Rio de Janeiro, Brazil; ^2^ Human Genetics Laboratory, Oswaldo Cruz Institute, Oswaldo Cruz Foundation, Rio de Janeiro, Brazil; ^3^ Postgraduate Program in Translational Biomedicine - UNIGRANRIO/AFYA, Duque de Caxias, Brazil; ^4^ Genetics Laboratory - UNIGRANRIO/AFYA, Duque de Caxias, Brazil; ^5^ Laboratory of Immunopharmacology, Oswaldo Cruz Institute, Oswaldo Cruz Foundation, Rio de Janeiro, Brazil

**Keywords:** endocannabinoid system, cannabinoid receptors, cancer biology, apoptosis, tumor angiogenesis

## Abstract

The Endocannabinoid System (ECS) plays a critical role in maintaining physiological homeostasis, influencing a range of processes such as neuroprotection, inflammation, energy metabolism, and immune responses. Comprising cannabinoid receptors (CB1 and CB2), endogenous ligands (endocannabinoids), and the enzymes responsible for their synthesis and degradation, the ECS has attracted increasing attention in cancer research. Cannabinoid receptor activation has been associated with the regulation of cancer-related processes, including cell proliferation, apoptosis, and angiogenesis, suggesting that the ECS may have a role in tumor progression and cancer treatment. Preclinical studies have shown that cannabinoids, through their interaction with CB1 and CB2 receptors, can inhibit tumor cell growth, induce programmed cell death, and suppress the formation of new blood vessels in various cancer models. Despite these encouraging findings, the clinical translation of ECS-targeted therapies remains in its early stages. The complexity of tumor heterogeneity, the variability in patient responses, and the challenges associated with the pharmacokinetics of cannabinoids are significant obstacles to the broader application of these findings in clinical settings. This review provides an overview of the current understanding of the ECS’s involvement in cancer biology, focusing on key mechanisms by which it may influence carcinogenesis. Additionally, we discuss the therapeutic potential of targeting the ECS in cancer treatment, while highlighting the limitations and uncertainties that need to be addressed through ongoing research.

## 1 Introduction

### 1.1 Contextualization of the endocannabinoid system in human biology

The Endocannabinoid System (ECS) is a sophisticated cellular signaling pathway crucial for maintaining physiological homeostasis. Identified in the early 1990s, the ECS comprises three main components: cannabinoid receptors, endocannabinoids, and the enzymes responsible for their synthesis and degradation (1, 2).

**FIGURE 1:**
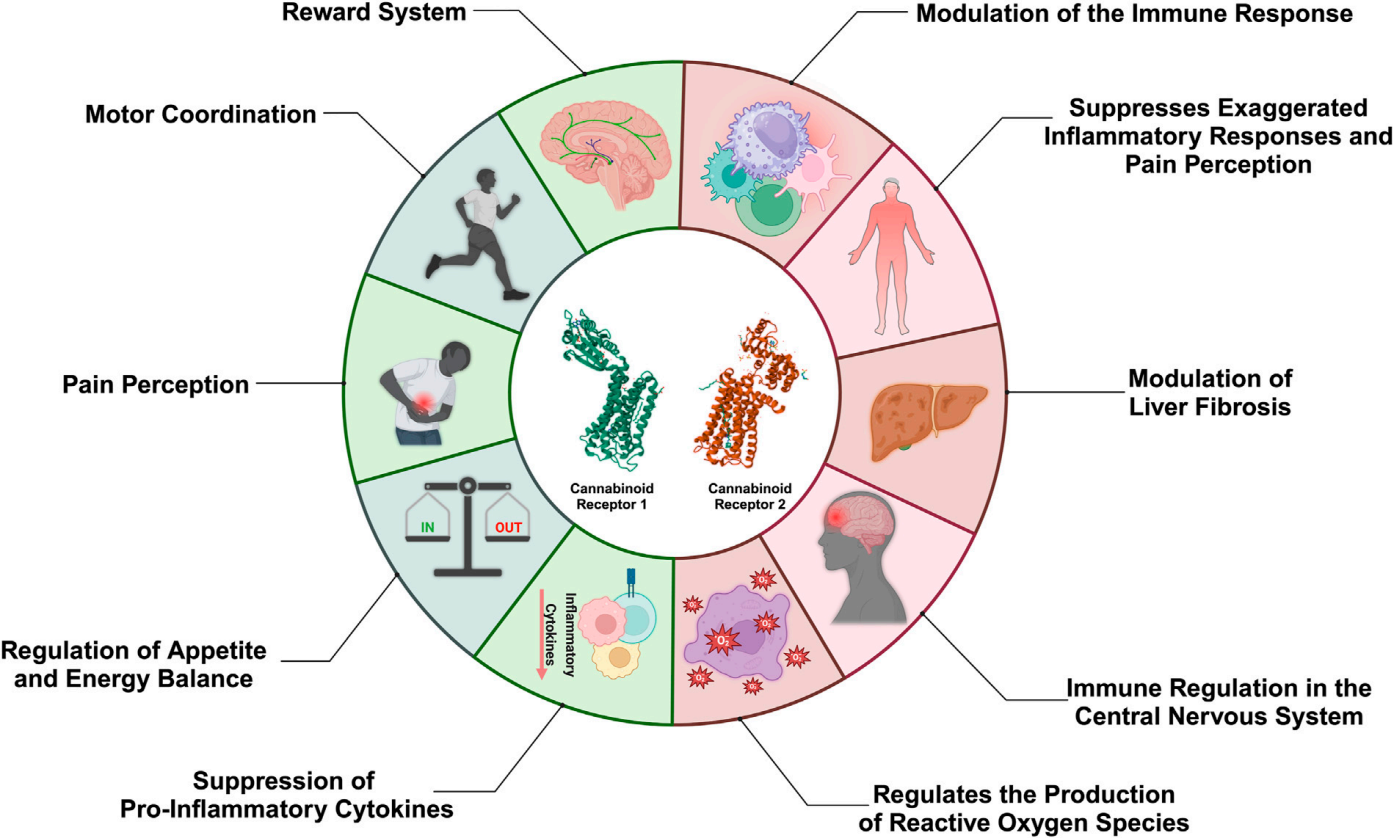
Schematic representation highlighting the diverse physiological roles of cannabinoid receptors CB1 and CB2. CB1, primarily distributed within the central nervous system, modulates reward pathways, motor coordination, pain perception, appetite regulation, and energy homeostasis, alongside exerting anti-inflammatory actions. Meanwhile, CB2, predominantly expressed in immune cells, regulates immune responses, mitigates excessive inflammation, influences liver fibrosis, and governs immune function within the central nervous system, in addition to controlling the production of reactive oxygen species (ROS). These functions underscore the pivotal involvement of CB1 and CB2 in both neurological and immunological processes, with ongoing investigations poised to reveal further intricacies and therapeutic potentials associated with these receptors.

Cannabinoid receptors CB1 and CB2, which belong to the G protein-coupled receptor family, play distinct and essential roles. CB1 receptors are widely distributed in the central nervous system and are responsible for modulating neurotransmitters that influence memory, motor coordination, and pain perception (3), while CB2 receptors are predominantly found in immune system cells such as macrophages and lymphocytes. They play a crucial role in modulating immune and inflammatory responses (4) (Figure 1).

In the context of intracellular signaling, CB1 and CB2 receptors initiate distinct but complementary pathways upon activation. When CB1 receptors are bound by endogenous ligands or synthetic agonists, they primarily couple with Gi/o proteins. This coupling leads to the inhibition of adenylate cyclase, a reduction in cyclic AMP levels, and subsequent modulation of ion channels. Ultimately, this cascade affects neurotransmitter release, contributing to the regulation of synaptic plasticity and neuroprotection (5). Conversely, when CB2 receptors are activated, mainly in immune cells, they engage Gi proteins to inhibit adenylate cyclase. In addition, they activate MAP kinase pathways, such as ERK1/2, which play a crucial role in regulating cellular proliferation, differentiation, and cytokine production (6).

**FIGURE 2:**
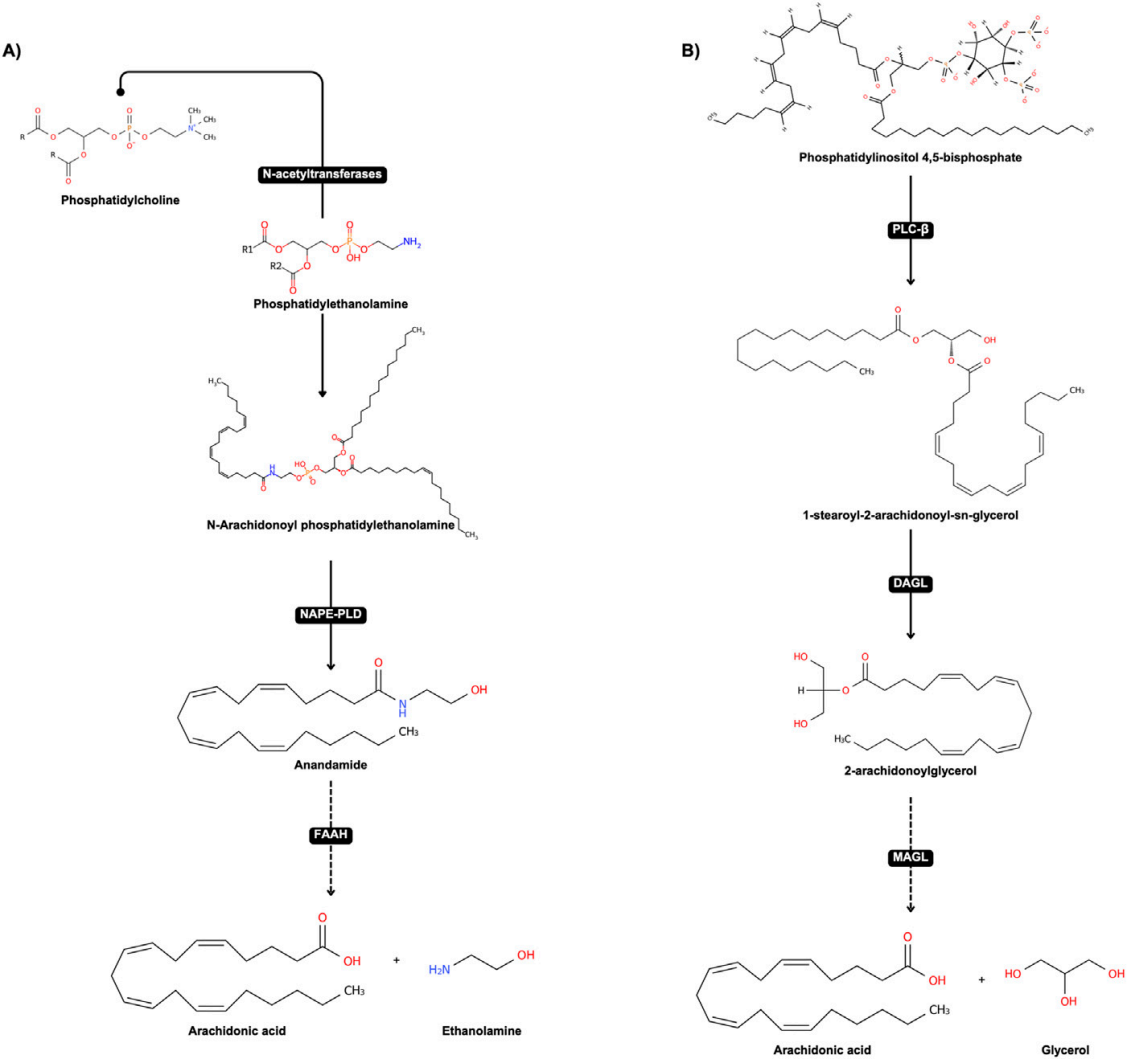
Classical pathways involved in the biosynthesis and degradation of endocannabinoids Anandamide (AEA) and 2-Arachidonoylglycerol (2-AG). **(A)** Biosynthesis and degradation of AEA: The biosynthesis of AEA begins with the transfer of an acyl group (commonly arachidonic acid) from Phosphatidylcholine (PC) to Phosphatidylethanolamine (PE), catalyzed by an N-acyltransferase, resulting in the formation of N-Arachidonoyl phosphatidylethanolamine (NAPE). Subsequently, NAPE is hydrolyzed by NAPE-specific phospholipase D (NAPE-PLD) to produce Anandamide (AEA). AEA is then degraded by fatty acid amide hydrolase (FAAH) into arachidonic acid and ethanolamine. **(B)** Biosynthesis and degradation of 2-AG: The biosynthesis of 2-AG involves the hydrolysis of Phosphatidylinositol 4,5-bisphosphate (PIP2) by phospholipase C-β (PLC-β), yielding diacylglycerol (DAG). DAG is then converted into 2-Arachidonoylglycerol (2-AG) by diacylglycerol lipase (DAGL). Finally, 2-AG is degraded by monoacylglycerol lipase (MAGL) into arachidonic acid and glycerol.

The primary endocannabinoids, anandamide (AEA) and 2-arachidonoylglycerol (2-AG) are derived from membrane lipids (Figure 2) and act as natural ligands for cannabinoid receptors. Anandamide, discovered by Devane et al. in 1992, is synthesized from N-arachidonoyl phosphatidylethanolamine (NAPE) and degraded by the enzyme fatty acid amide hydrolase (FAAH) (1). 2-AG, identified by Mechoulam et al. in 1995, is produced from diacylglycerol (DAG) and degraded by monoacylglycerol lipase (MAGL) (2).

The ECS regulates crucial processes such as neuroprotection, appetite control, pain regulation, and immune response modulation. Alterations in endocannabinoid signaling are associated with various pathological conditions, including neurodegenerative diseases (e.g., Alzheimer’s and Parkinson’s), chronic inflammation, obesity, and cancer (7). Recent studies highlight that the ECS also plays a vital role in oncogenesis and tumor progression, with cannabinoid receptor activation showing the potential to inhibit cell proliferation, induce apoptosis, and reduce tumor angiogenesis, suggesting the ECS as a promising pathway for cancer therapies (8, 9).

#### 1.1.1 Importance of the ECS in regulating physiological processes

In the central nervous system, CB1 receptors regulate neurotransmission, affecting neurotransmitters such as glutamate, dopamine, and GABA release. This regulation impacts synaptic plasticity, memory, learning, and pain perception (10, 11). Studies have shown cannabinoid receptor activation can reduce pain sensitivity in acute and chronic contexts (12, 13). In the immune system, CB2 receptors are fundamental for modulating the inflammatory response. Activation of these receptors in immune cells, such as macrophages and lymphocytes, leads to reduced production of pro-inflammatory cytokines and promotes an anti-inflammatory state, which is beneficial in diseases such as rheumatoid arthritis and multiple sclerosis (14, 15).

The ECS also plays an important role in energy metabolism and appetite control. CB1 receptors in the hypothalamus stimulate appetite, while endocannabinoids such as anandamide and 2-AG participate in satiety signaling and energy balance (16–18). In the cardiovascular system, the ECS can influence blood pressure and cardiac function. Studies suggest that endocannabinoids promote vasodilation and protect the heart during ischemia/reperfusion events, highlighting a significant cardioprotective role (19).

In addition to these functions, the ECS is extensively studied in cancer. Cannabinoid receptor activation is associated with antiproliferative, pro-apoptotic, and anti-angiogenic effects in tumor cells. For instance, activation of CB1 and CB2 receptors in glioma cells inhibits cell proliferation and induces apoptosis (20). Moreover, the ECS can modulate tumor angiogenesis by inhibiting the expression of pro-angiogenic factors such as VEGF and reducing tumor vascularization (21, 22).

Thus, the regulation of physiological processes by the ECS is multifaceted, and its proper functioning is critical for bodily homeostasis. Dysfunction of this system can lead to a variety of pathological conditions, including cancer, reinforcing the importance of a thorough understanding of the ECS for developing new therapeutic approaches.

## 2 Introduction to cancer: global prevalence and impact

Cancer remains one of the leading causes of morbidity and mortality worldwide. In 2020, the Global Cancer Observatory (GCO) recorded 19,976,499 new cases, with lung, breast, colorectal, prostate, stomach, and liver cancers being the most common. Cancer mortality is also alarming, with 9,743,832 deaths in the same year. The deadliest cancers include lung, colorectal, liver, breast, stomach, and pancreatic cancers (23).

Cancer distribution varies significantly among continents. For example, Asia accounted for 49.2% of global incidence cases and a mortality rate of 56.1%. Europe reported 22.4% of incidence cases, with a mortality rate of 20.4%. North America had 13.4% of incidence cases and 7.2% of the global mortality rate. Latin America and the Caribbean accounted for 7.8% of incidence cases and 7.7% of deaths. Africa had 5.9% of incidence cases and a mortality rate of 7.8%. Oceania had 1.3% of incidence cases and 0.76% of deaths (23).

Sex-based differences are also notable. Lung, breast, and colorectal cancers are the most common globally, but prevalence varies between males and females. Prostate cancer is exclusive to males, while breast cancer is more prevalent in females. Age-adjusted incidence rates are higher in men (289.9–514.3 per 100,000) than in women (231.8–415.2 per 100,000). Mortality is also higher among men (197.0–289.9 per 100,000) compared to women (176.2–231.8 per 100,000), with lung, liver, and stomach cancers predominating among men (23).

This chapter aims to explore the interaction between the ECS and cancer, elucidating how endocannabinoids influence carcinogenesis and highlighting advancements in preclinical research suggesting potential therapeutic applications.

### 2.1 Cancer biology

#### 2.1.1 Overview of carcinogenesis processes

Carcinogenesis is a multi-phase process that transforms normal cells into malignant ones through genetic and epigenetic alterations. This process is traditionally divided into three stages: initiation, promotion, and tumor progression.

During initiation, permanent genetic mutations are introduced into cellular DNA, often induced by chemical carcinogens, ionizing radiation, or viral infections. These mutations typically affect oncogenes, tumor suppressor genes, and genes involved in DNA repair, conferring a proliferative advantage to the transformed cells (24). In the promotion phase, initiated cells begin to proliferate in response to stimuli such as chronic inflammation or hormonal imbalances. Although no new genetic mutations occur, these pre-malignant cells expand clonally, acquiring characteristics that favor tumor progression. Promoting factors, including hormones and inflammatory mediators, facilitate cellular proliferation without directly inducing DNA damage (25). The progression phase is characterized by the acquisition of additional malignant properties, such as tissue invasion and metastasis. During this stage, tumor cells accumulate additional mutations and epigenetic changes that promote immune system evasion, resistance to apoptosis, and angiogenesis induction. Additionally, epithelial-to-mesenchymal transition (EMT) significantly contributes to tumor invasiveness and metastatic dissemination (26).

Advances in understanding carcinogenesis mechanisms have driven the development of targeted therapies and immunotherapies, resulting in significant improvements in clinical outcomes. In particular, research into the ECS has revealed its emerging therapeutic potential, affecting critical processes of carcinogenesis such as cell proliferation, apoptosis, and angiogenesis, and offering new perspectives for specific therapeutic strategies (27).

## 3 Effects of the endocannabinoid system on cancer biology: cell proliferation, cell death, and tumor angiogenesis

The Endocannabinoid System (ECS) has been extensively studied for its impact on cancer biology, particularly in critical processes such as cell proliferation, apoptosis, and angiogenesis. Evidence from both *in vitro* and *in vivo* preclinical studies (Supplementary Table S1) highlights the modulatory role of the ECS in these key mechanisms of carcinogenesis. It is important to note that the antitumor effects of the endocannabinoid system are driven by both endogenous endocannabinoids and exogenous phytocannabinoids like Δ9-tetrahydrocannabinol (Δ9-THC) and cannabidiol (CBD), each with distinct pharmacological properties. Endocannabinoids primarily activate CB1 and CB2 receptors, while phytocannabinoids also target GPR55, TRPV1, and PPARγ. For example, Δ9-THC, a partial agonist of CB1/CB2, induces apoptosis by inhibiting the PI3K/AKT pathway, whereas CBD, with low affinity for these receptors, activates TRPV1 and antagonizes GPR55, causing endoplasmic reticulum stress and cell death (28, 29).

Cell Proliferation: Activation of CB1 and CB2 receptors has been shown to inhibit tumor cell proliferation in various types of cancer. For instance, a 2020 study demonstrated that CBD significantly reduces the viability and proliferation of pancreatic cancer cells. This action was associated with inhibiting the p-21-activated kinase (PAK1) signaling pathway. The study suggests that CBD exerts antitumor effects by modulating the oncogene Kras pathway, specifically targeting PAK1 and contributing to tumor growth inhibition (30). Additionally, an *in vivo* study using a mouse model of glioblastoma revealed that the combination of Δ9-THC and CBD not only reduced tumor growth through mitochondrial damage and disruption of energy metabolism but also improved animal survival, suggesting a synergistic effect between these phytocannabinoids (31). Beyond phytocannabinoids, the endocannabinoid 2-AG has been shown to suppress proliferation in prostate cancer models through downregulation of cyclin-dependent kinases (CDKs) (21).

Apoptosis: Induction of apoptosis by Δ9-THC has been documented in human colorectal cancer cells, where the compound inhibited the RAS-MAPK and PI3K-AKT survival signaling pathways and activated the pro-apoptotic protein BAD. This effect involved the production of reactive oxygen species (ROS) and activation of caspases, key enzymes in apoptosis execution (32). Similarly, a 2023 study demonstrated that CBD induces apoptosis and macroautophagy in colorectal cancer cells, mediated by proteins p53 and Hsp70. CBD administration resulted in increased ROS production and caspase activation, promoting cell death. The inhibition of Hsp70 also intensified apoptosis, suggesting that CBD could be a promising therapeutic approach for colorectal cancer (33). Endocannabinoids also exert pro-apoptotic effects. AEA induces apoptosis in ovarian cancer cells via ceramide accumulation and caspase activation (34), while 2-AG promotes cell death in glioblastoma through suppression of the anti-apoptotic protein Bcl-2 (20).

Angiogenesis: Angiogenesis is crucial for tumor growth and dissemination, and the ECS has been shown to modulate this process. An *in vitro* study assessed the impact of CBD on colorectal cancer-associated angiogenesis, revealing that CBD inhibited new blood vessel formation by reducing the expression of vascular endothelial growth factor (VEGF) and pro-angiogenic cytokines in tumor cells (35). AEA and 2-AG modulate tumor angiogenesis: AEA suppresses VEGF expression in breast cancer (22), and 2-AG inhibits endothelial cell migration in hepatic models (21). Additionally, administration of the selective CB2 receptor agonist, JWH133, in a mouse glioma model significantly reduced tumor angiogenesis and glioma growth, suggesting that ECS modulation could be an effective strategy to limit cancer progression (36).

### 3.1 Clinical evidence of cannabinoids in cancer treatment

Cannabinoids, substances derived from the *Cannabis sativa* plant, have demonstrated potentially anticancer properties in laboratory studies by targeting cancer cells while protecting healthy tissues. These effects occur through interactions with specific receptors or distinct pathways, with efficacy varying based on cancer type and cannabinoid concentration (37). Despite promising preclinical findings, clinical studies on the efficacy of cannabinoids in human cancer treatment remain limited. Evidence suggests that cannabinoids may alleviate symptoms such as nausea, pain, and appetite loss associated with cancer, and are generally well-tolerated, though side effects can vary depending on the compound used (38). While cannabinoids are well-supported in palliative oncology care, further research is essential to explore their anticancer potential and establish optimal dosing and administration strategies (37, 39).

However, translating the therapeutic potential of the Endocannabinoid System (ECS) into clinical applications for cancer treatment presents significant challenges. Preclinical models, including cell cultures and animal studies, often fail to fully represent the complexity of human tumors. Physiological barriers, such as limited tumor penetration and intratumoral heterogeneity, reduce the efficacy of treatments that initially show promise in laboratory settings (40, 41).

Another key obstacle is the emergence of subclonal resistance mutations within tumor cell populations. These genetic alterations diminish the durability of therapeutic responses and complicate the development of effective ECS-based therapies. As Schmitt et al. note, genetic diversity and resistant subpopulations pose significant challenges to identifying reliable molecular targets for therapy (42).

Furthermore, discrepancies between preclinical and clinical outcomes highlight the need for more predictive models. Advanced approaches, such as organoids and patient-derived xenografts, offer more accurate representations of the human tumor microenvironment and may help reduce high failure rates observed in early clinical trials (43).

Finally, the development of ECS-based therapies is hindered by high costs and lengthy development timelines. Adaptive clinical trial designs and closer integration between preclinical and clinical research could accelerate the progress of ECS-targeted cancer treatments (44, 45).

### 3.2 Cannabinoid mechanisms of action and the impact of tumor heterogeneity

The ECS has increasingly become a target of research linking its regulation to potential tumor control. CB1 expression is elevated in prostate tumors, for example. ECS receptors not only promote apoptosis in prostate cancer cells and limit the migration and invasion of this tumor but also facilitate the interaction of ECS substances to control tumor cell growth and proliferation. The substances used to regulate the ECS in prostate cancer are also employed in other tumors and may have similar or entirely different effects depending on the types of cells in the different tumors studied (46).

Similarly, in breast cancer, one of the leading causes of cancer-related mortality among females, endocannabinoids and their exogenous analogs, such as THC, exhibit antitumor effects in various animal cancer models. However, their effects in breast cancer are complex, with evidence indicating both antitumor and protumor roles depending on the biological context. This duality underscores the need for further investigation to clarify the role of the ECS in breast cancer progression and therapy (47).

In lung cancer, cannabinoids have demonstrated significant therapeutic potential as adjunctive treatments. Studies show their ability to inhibit lung cancer cell viability, induce apoptosis, and modify the tumor microenvironment to reduce proliferation, migration, invasion, angiogenesis, and metastasis, while providing additional therapeutic benefits (48).

Despite these promising findings, tumor heterogeneity remains a major barrier to effective cancer therapies, including ECS-based treatments. Tumor heterogeneity is characterized by the coexistence of distinct subpopulations of cells with varying genetic, epigenetic, and molecular profiles within the same tumor (intratumoral heterogeneity) or among tumors of the same subtype (intertumoral heterogeneity). This diversity drives treatment resistance and tumor progression by fostering a dynamic microenvironment where subclones adapt to selective pressures, including therapeutic interventions. In solid tumors like breast, lung, and prostate cancers, intratumoral variability is further amplified by clonal evolution, phenotypic plasticity, and interactions with the tumor microenvironment, presenting significant challenges to therapeutic efficacy (49–51).

In this challenging landscape, the ECS offers a multifaceted approach to therapeutic intervention. Studies have shown that the ECS modulates tumor progression through complex mechanisms, with effects dependent on cell type, cannabinoid receptor activation (CB1 and CB2), and the surrounding microenvironment. CB2, primarily expressed in immune cells, has demonstrated potential to limit inflammatory processes and modulate immunosuppression in the tumor microenvironment, thereby acting as a barrier against metastasis. Conversely, CB1, which is widely expressed in the central nervous system and various neoplasms, may induce apoptosis or support cell survival, depending on the molecular and cellular context (28, 29, 52, 53, 54).

Further complicating this picture is the discovery of additional ECS-related receptors, such as GPR55 and TRPV1, which form functional heteromers with CB1 and CB2. These interactions create tumor-specific signaling circuits, allowing the ECS to influence critical processes in tumor biology, including angiogenesis, cell migration, proliferation, and apoptosis. In tumors with high heterogeneity, the pleiotropic effects of cannabinoids vary widely across subpopulations, complicating the prediction of uniform therapeutic responses (28, 50). Emerging technologies, such as single-cell sequencing and liquid biopsies, are now enabling the identification of tumor subclones that are either responsive or resistant to ECS modulation, paving the way for more personalized treatment approaches (28).

Understanding the interplay between tumor heterogeneity and ECS modulation is essential for developing therapies that reduce tumor burden while preventing resistance and recurrence. Integrating these insights into treatment planning offers a unique opportunity to advance precision oncology. By combining the therapeutic potential of the ECS with a detailed understanding of tumor architecture, it becomes possible to redefine therapeutic strategies and significantly improve outcomes for cancer patients (29, 49, 51).

### 3.3 Patient variability and personalized approaches in ECS-Targeted therapies

The variability in patient responses to ECS-targeted therapies underscores the importance of adopting personalized approaches in oncology. Evidence suggests that genetic, epigenetic, and phenotypic factors, along with tumor microenvironment characteristics, significantly impact the efficacy and tolerability of these treatments, even among patients with similar biomarker profiles (55, 56). Differences in signaling pathways, receptor expression (CB1 and CB2), and the presence of specific mutations further emphasize the necessity of tailoring cannabinoid-based therapies to the molecular characteristics of each patient to optimize therapeutic outcomes and minimize adverse effects (28, 29).

The incorporation of predictive biomarkers into clinical trial designs has been shown to improve therapeutic efficacy. Meta-analyses of phase II trials have demonstrated that personalized approaches result in higher response rates and prolonged progression-free survival compared to non-personalized strategies (57, 58). Within the context of ECS modulation, biomarkers can guide the selection of specific agonists or antagonists.

Personalized medicine, as applied to ECS therapies, utilizes biomarkers to align treatment strategies with the biological profile of individual patients. For example, a personalized medicine initiative at the MD Anderson Cancer Center demonstrated that patients receiving biomarker-matched therapies in phase I clinical trials exhibited significantly improved response rates and extended time to treatment failure compared to those receiving non-matched therapies (45, 59). These findings highlight the potential of individualized treatment models in enhancing therapeutic precision, particularly within the complex framework of the ECS.

In conclusion, understanding the factors that influence patient variability in ECS-targeted treatments is essential for advancing more effective therapeutic strategies. The integration of predictive biomarkers and patient phenotyping into clinical trial designs represents a crucial step toward precision oncology, enabling tailored treatments that address the inherent complexity of cancer on an individual basis (60, 61).

## 4 Therapeutic challenges and considerations

Cannabidiol has low bioavailability, variable pharmacokinetic profiles, and potential polymorphisms, which may lead to unpredictable efficacy, as well as increased side effects and drug interactions. Furthermore, new formulations, such as self-emulsifying drug delivery systems and improved crystalline structures, are being developed to overcome the challenges of cannabidiol delivery. These new formulations appear promising in improving the bioavailability and pharmacokinetic profiles of CBD (62).

Regulation of cannabinoid use in treatments varies widely between countries and regions. In the European Union, although there is a common framework for authorized medicines, the regulation of authorized preparations containing cannabinoids is according to the criteria of each Member State, leading to uneven access (63). As of 2017, only four countries (Canada, Germany, Israel, and the Netherlands) had fully authorized the medical use of herbal cannabis, with most regulatory agencies allowing physicians to determine specific indications (64). Cannabinoids have shown potential in cancer treatment, especially in palliative care, but their antitumor effects depend on the type of tumor and dose (65). Currently, approved medications target specific conditions such as chemotherapy-induced nausea and appetite stimulation (66).

## 5 Legal and ethical aspects

The use of cannabinoids in cancer treatment raises complex ethical and regulatory challenges, particularly due to gaps in registration and certification processes (67). While evidence of their efficacy in cancer therapy remains limited, cannabinoids have demonstrated potential in managing symptoms such as neuropathic pain, nausea, and sleep disorders. Their multimodal action and relatively favorable side effect profile make them an attractive option, especially for patients in palliative care (68). However, the limited knowledge among healthcare professionals regarding cannabinoids complicates patient education and treatment monitoring (38, 68).

Legal disparities significantly impact access to cannabinoid-based therapies. Regulatory frameworks vary widely between countries and even within states, resulting in unequal availability for patients. While some regions have established clear guidelines, others maintain restrictive policies, excluding many from potential benefits (54, 65). These inconsistencies not only limit patient access but also leave healthcare providers uncertain about prescribing practices, often due to fears of legal repercussions (69).

Ethical concerns are further complicated by the variability in therapeutic responses to cannabinoids, which depend on cancer type, dosage, and individual patient profiles. Cannabinoids may induce tumor regression in some cases but have the potential to promote tumor growth in others, highlighting the need for robust clinical evidence to guide their safe and effective use (70, 71). This variability necessitates personalized treatment approaches while raising concerns about equitable access to such individualized care (69).

Patient autonomy is another critical consideration. Many oncology patients seek cannabinoid-based therapies to improve quality of life, yet the social stigma and limited availability of scientific information often hinder informed decision-making. Additionally, many healthcare professionals lack sufficient training to effectively educate patients about these treatments (38). Transparent communication about the risks and benefits of cannabinoids is essential to respect patient autonomy while adhering to the principles of beneficence and non-maleficence.

The potential neurotoxic and psychoactive effects of cannabinoids, particularly THC, present unique ethical challenges for vulnerable populations such as pediatric and geriatric patients. Long-term effects, especially on neurocognitive development in children, remain poorly understood, underscoring the need for rigorous research and guidelines to ensure patient safety (70). Regulatory oversight of product purity, potency, and dosing is critical to mitigate these risks and support ethical clinical practices.

Policy-related barriers also hinder progress in cannabinoid therapies. Limited access to high-quality study-grade compounds, insufficient research funding, and the slow pace of policy reform restrict advancements in the field (72). Although recent legislative changes in certain regions have expanded opportunities for research and clinical applications, they have also introduced concerns about commercialization and disparities in access to these therapies (73).

Addressing the ethical and legal challenges of ECS-targeted therapies requires a cohesive strategy. A clear regulatory framework and robust research, alongside equitable access, are vital for the safe use of cannabinoids in oncology. By bridging gaps in policy and education, we can realize the therapeutic potential of cannabinoids while ensuring patient wellbeing and fairness in cancer care.

## 6 Conclusion and future directions

The ECS is emerging as a significant component in cancer biology, offering new possibilities for therapeutic intervention. Laboratory studies demonstrate that activating ECS receptors can influence critical aspects of cancer progression, including cell proliferation, apoptosis, and angiogenesis. These findings highlight the potential of ECS modulation to slow tumor growth and pave the way for novel treatments. However, translating these insights into effective and accessible therapies remains challenging due to the variability in cannabinoid effects across cancer types and individual patient responses. Personalized approaches will likely be essential to maximize therapeutic efficacy while minimizing risks.

The role of the ECS in modulating the TME, therapeutic resistance, and immune responses positions it as a promising target for advancing oncology. CB1 and CB2 receptors interact with key signaling pathways, such as PI3K/AKT/mTOR, to regulate angiogenesis, cell invasion, and immune modulation. These interactions make the ECS a viable avenue for overcoming resistance mechanisms, particularly in tumors with high heterogeneity or stem cell-mediated resistance (74–76).

Combination therapies involving cannabinoids and conventional approaches, such as chemotherapy and radiotherapy, have shown synergistic effects. For instance, THC induces apoptosis and modulates endoplasmic reticulum stress, while CBD inhibits tumor cell proliferation and metastasis with low toxicity in preclinical models. Cannabinoids also enhance the efficacy of immunotherapies by modulating immune cell activity within the TME (75, 77, 78). These findings suggest that integrating cannabinoids into combination regimens could improve therapeutic outcomes across various cancer types.

The application of genomic technologies, such as NGS, further supports the personalization of ECS-based therapies. These tools enable the identification of mutations and biomarkers associated with ECS pathways, facilitating patient stratification and the development of tailored treatment strategies. Advanced preclinical models, including 3D organoids and lab-on-chip devices, provide robust platforms to simulate the complexity of the TME and study cannabinoid effects under physiologically relevant conditions (78–80).

Despite these advancements, significant economic and regulatory barriers must be addressed to integrate ECS-based therapies into clinical practice. Rigorous clinical trials, cost-effectiveness evaluations, and clear regulatory frameworks are crucial to ensure accessibility, safety, and efficacy. Recent studies emphasize the importance of proper regulation and implementation strategies to democratize access, particularly in healthcare systems constrained by economic limitations (79, 80).

In summary, the ECS represents a promising avenue in cancer treatment, with the potential to address some current limitations and contribute to advancements in personalized medicine. While significant challenges remain, including the need for robust clinical evidence, improved regulatory frameworks, and greater accessibility, continued research and integration of emerging technologies may enable ECS-based therapies to complement existing treatments and offer more tailored options for cancer patients.
