# Supplementary material for: The endocannabinoid system in cancer biology: a mini-review of mechanisms and therapeutic potential
Source: Oncol Rev. 2025 Apr 30;19:1573797. doi: 10.3389/or.2025.1573797 (PMC12075236; doi:10.3389/or.2025.1573797)
Supplement: Supplementary file 1 [file Table1.DOCX]

Supplementary Material

# Supplementary Table

**Supplementary Table 1.** Clinical Trials Involving the Endocannabinoid System

| **Trial ID** | **Clinical Phase** | **Cannabinoid Type** | **Indication (Cancer Type)** | **Status** |
| --- | --- | --- | --- | --- |
| NCT05629702 | Phase 2 | CBD | Methylated Glioblastoma | Recruiting |
| NCT06533657 | Phase 2 | Δ9-THC + CBD | Cancer Pain | Not yet recruiting |
| NCT05520294 | Observational | CBD | Malignant Melanoma | Active, not recruiting |
| NCT05272865 | Phase 1/2 | Δ9-THC | Post-Chemotherapy Patients | Not yet recruiting |
| NCT00314808 | Phase 1 | CBD | Primary Gliomas | Completed |
| NCT02255292 | Phase 2 | Δ9-THC + CBD | Solid Tumor | Status unknown |
| NCT01812603 | Phase 1/2 | Δ9-THC + CBD | Recurrent Glioblastoma | Completed |
| NCT01654497 | Phase 1 | CBD | Brain Cancer | Completed |
| NCT03607643 | Phase 1/2 | CBD | Multiple Myeloma | Status unknown |
| NCT04001010 | Phase 3 | CBD | Advanced Cancer | Suspended |
| NCT03529448 | Phase 2 | Δ9-THC + CBD | Newly Diagnosed Glioblastoma | Active, not recruiting |
| NCT03984214 | Phase 3 | Δ9-THC | Pancreatic Cancer | Active, not recruiting |

CBD,Cannabidiol, a chemical compound derived from the cannabis plant; Δ9-THC, Delta-9-tetrahydrocannabinol, the primary psychoactive compound found in cannabis.
